# Design of Glucagon-Like Peptide-1 Receptor Agonist for Diabetes Mellitus from Traditional Chinese Medicine

**DOI:** 10.1155/2014/385120

**Published:** 2014-05-06

**Authors:** Hsin-Chieh Tang, Calvin Yu-Chian Chen

**Affiliations:** ^1^Department of Biomedical Informatics, Asia University, Taichung 41354, Taiwan; ^2^Department of Medicine, China Medical University, Taichung 40402, Taiwan

## Abstract

Glucagon-like peptide-1 (GLP-1) is a promising target for diabetes mellitus (DM) therapy and reduces the occurrence of diabetes due to obesity. However, GLP-1 will be hydrolyzed soon by the enzyme dipeptidyl peptidase-4 (DPP-4). We tried to design small molecular drugs for GLP-1 receptor agonist from the world's largest traditional Chinese medicine (TCM) Database@Taiwan. According to docking results of virtual screening, we selected 2 TCM compounds, wenyujinoside and 28-deglucosylchikusetsusaponin IV, for further molecular dynamics (MD) simulation. GLP-1 was assigned as the control compound. Based on the results of root mean square deviation (RMSD), solvent accessible surface (SAS), mean square deviation (MSD), Gyrate, total energy, root mean square fluctuation (RMSF), matrices of smallest distance of residues, database of secondary structure assignment (DSSP), cluster analysis, and distance of H-bond, we concluded that all the 3 compounds could bind and activate GLP-1 receptor by computational simulation. Wenyujinoside and 28-deglucosylchikusetsusaponin IV were the TCM compounds that could be GLP-1 receptor agonists.

## 1. Introduction

A new trend for management of obesity and diabetes mellitus (DM) has seen the light of dawn. One study has found the mechanism to lower glucose levels in diabetic patients and reduce their weight effectively [1]. DM is a worldwide disease and represents high blood sugar in the patients [2]. It is considered as a kind of modern disease [3]. The pathogenesis of DM is destruction of islet cells in pancreas [4]. Islet-cell antibodies are associated with the troublesome disease [5]. Human leukocyte antigen (HLA) gene contributes to insulin resistance [6, 7]. Defects in *β*-cell function are failure to secret insulin [8]. DM is often accompanied with hypertension and renal disease [9]. It is a member of metabolic syndrome [10]. DM can simply be divided into three main types: type 1 DM (insulin-dependent, IDDM), type 2 DM (noninsulin-dependent, NIDDM), and gestational DM [11]. Early diagnosis and adequate treatment are very important for progression of the disease [12]. DM can cause many acute and chronic complications. Acute complications include diabetic ketoacidosis and even coma. Chronic complications include vascular diseases, such as coronary heart disease, retinopathy, and renal failure [13]. There are many risk factors for the annoying disease [14]. DM is related to incorrect diet and irregular life style [15]. Obesity is an increasing problem in many developed and developing countries [16]. DM and obesity are inseparable [17]. Excessive body mass index (BMI) increases the risk of DM [18].

Golden treatment of type 1 DM (IDDM) is injected insulin [19, 20]. Insulin resistance is main problem for type 2 DM (NIDDM). There are many predisposing mechanisms for type 2 DM [21]. Therapeutic agents for type 2 DM include increasing insulin secreted by the pancreas, increasing the sensitivity of target organs to insulin, and decreasing glucose uptake from the gastrointestinal tract [22]. Sulfonylureas have the ability to increase insulin secreted by the pancreas [23]. Metformin and thiazolidinediones increase the sensitivity of target organs to insulin [24, 25]. Acarbose can slow glucose uptake from the gastrointestinal tract [26]. However, most of above agents have side effects. The most threatening side effect is cardiovascular problem [27]. Thiazolidinediones are the notorious representatives [28, 29].

A new, advancing agent for management of DM is coming [30]. Incretin is the member of gastrointestinal hormones [31]. This hormone can decrease blood glucose level [32]. Type 2 DM can be treated by incretin injection [33]. Incretin-based therapies have been applied in this type of DM successfully [34]. The typical incretin is glucagon-like peptide 1 (GLP-1) [35]. Food can improve GLP-1 secretion in the intestine [36]. GLP-1 has antidiabetic effect through many mechanisms. It increases expression of the pancreatic beta cell receptors [37]. The incretin receptor belongs to G protein-coupled receptors [38]. It can increase insulin biosynthesis in the pancreas. By the other way, it can decrease glycogen release in the liver. It can also lower appetite in the brain and inhibit gastric emptiness in the stomach [39–41]. However, GLP-1 will be hydrolyzed soon by the enzyme dipeptidyl peptidase-4 (DPP-4) [42].

Due to modern technology in medicine, we describe the mechanism of several diseases [43–45]. Some diseases could not explain in the past days, but we can explore them by new biomedical methods now [46–48]. A lot of therapies have emerged nowadays [49–51]. Better life quality is no longer impossible in the future [52–54]. GLP-1 is a promising target for DM therapy and reduces the occurrence of diabetes due to overweight or obesity. Thus it is possible that we can design appropriate drugs to be GLP-1 receptor agonist. Computer-aided drug design (CADD) is a time-saving method to filter large amounts of small compounds by computational simulation [55]. CADD has been widely used in the forward-looking treatment of diseases [56, 57]. Through virtual screening of candidates and validation by molecular dynamics simulation techniques, we can design effective and novel drugs for many troubling diseases [58, 59]. Traditional Chinese medicine (TCM) has been considered as effective treatment for a lot of diseases [60, 61]. We tried to design suitable small molecular drugs for GLP-1 receptor agonist based on the world's largest TCM Database@Taiwan in this study [62].

## 2. Materials and Methods

### 2.1. Data Collection

We employed the TCM Database@Taiwan (http://tcm.cmu.edu.tw/) from which all small molecular compounds were downloaded to identify potential GLP-1 receptor agonist screening [62]. All TCM compounds were verified by Lipinski's rule of five [63]. The GLP-1 receptor protein sequence was acquired from the Uniprot Knowledgebase (P43220, human). The 3D structure of human GLP-1 receptor was acquired from Protein Data Bank (PDB ID: 3C5T).

### 2.2. Structure-Based Virtual Screening

The ligands from TCM Database@Taiwan and the control (GLP-1) were conducted for docking with GLP-1 protein. We utilized the LigandFit module in DS 2.5 to perform docking procedure. All docking poses were minimized by the force field of Chemistry at HARvard Molecular Mechanics (CHARMm). We calculated the scores of piecewise linear potentials (-PLP), potential of mean force (-PMF) by the LigandFit module in DS 2.5. LIGPLOT program was adopted to illustrated hydrogen bond (H-bond) and hydrophobic contact between the ligand and protein [64, 65].

### 2.3. Disorder Prediction

We utilized the program of PONDR-FIT in the DisProt website to exclude the disordered residues of 3D structure of GLP-1 receptor [66, 67].

### 2.4. Molecular Dynamics (MD) Simulation

We employed the package of GROningen MAchine for Chemical Simulations (GROMACS) for MD simulation. Four phases for selected protein-ligand complex were minimization, heating, equilibration, and production. The trajectory analytic figures of root mean square deviation (RMSD), solvent accessible surface (SAS), mean square deviation (MSD), Gyrate, total energy, root mean square fluctuation (RMSF), matrices of smallest distance of residues, database of secondary structure assignment (DSSP), and cluster analysis were drawn to explore the secret of MD simulation. We illustrated ligand corresponding protein change and GLP-1 receptor protein alone to compare the difference of binding during MD. Distance of H-bond between the ligand and essential amino acids was calculated too. Best distance of H-bond was set at 0.3–0.35 nm [68].

### 2.5. Ligand Pathway

We utilized the CAVER software to analyze all possible ligand pathways when the ligand bound with GLP-1 receptor [69].

## 3. Results and Discussion

### 3.1. Structure-Based Virtual Screening

**Figure 1:**
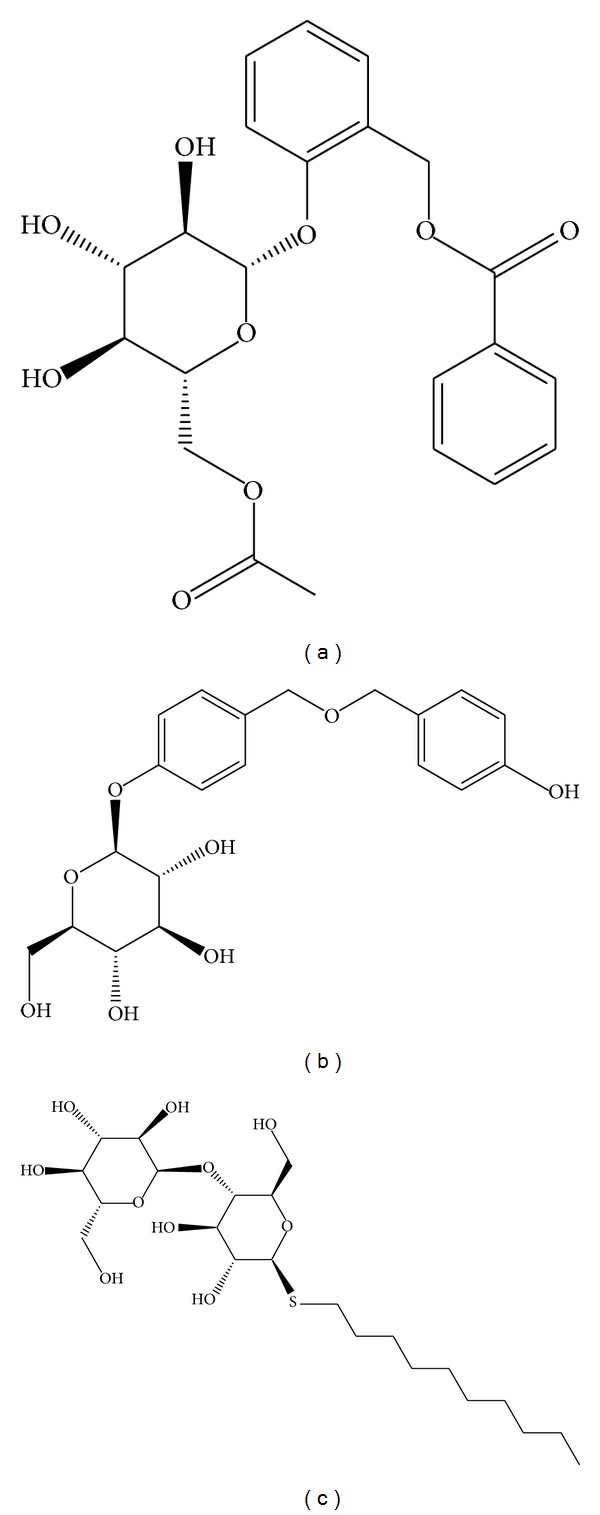
Scaffold of top 2 TCM candidates: (a) wenyujinoside, (b) 28-deglucosylchikusetsusaponin IV, and the control: (c) glucagon-like peptide 1 (GLP1).

**Figure 2:**
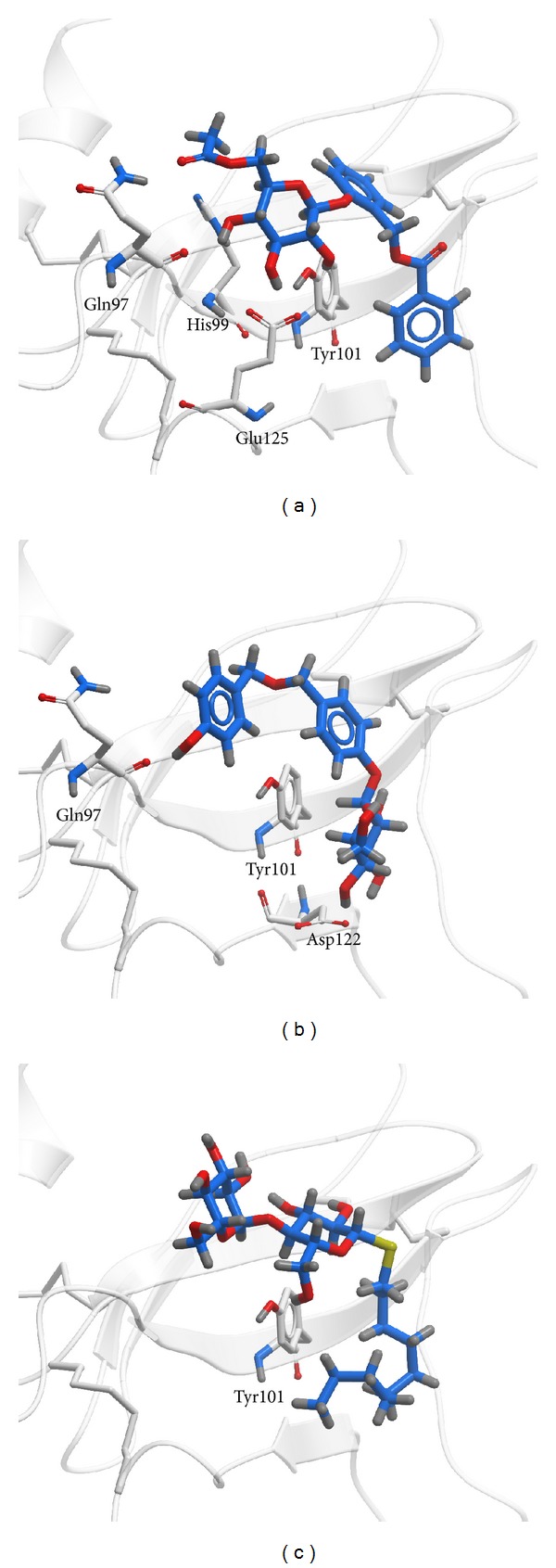
Docking poses by the LigandFit module in DS 2.5. (a) Wenyujinoside, (b) 28-deglucosylchikusetsusaponin IV, and the control: (c) GLP1.

**Figure 3:**
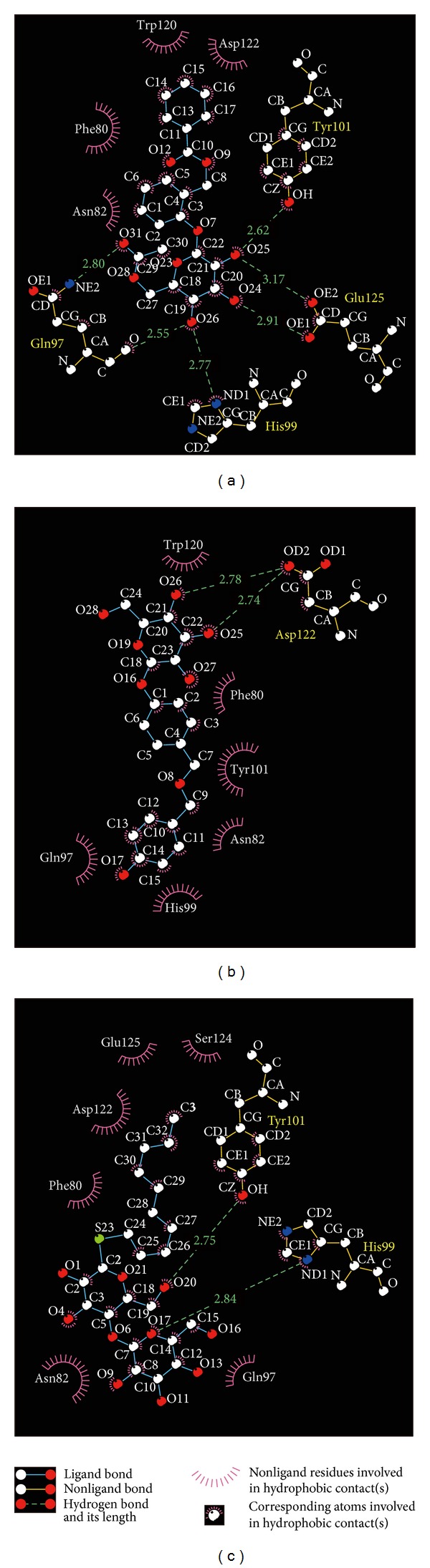
Docking poses by the LIGPLOT program. (a) Wenyujinoside, (b) 28-deglucosylchikusetsusaponin IV, and the control: (c) GLP1.

Table 1 listed -PLP2, -PLP1, and -PMF of the top 10 TCM compounds ranked by -PLP2. -PLP1 or -PLP2 was one type of dock score that evaluated the atom types of ligand and receptor. The difference of -PLP2 from -PLP1 was that an atomic radius was assigned to each atom. Integrating these data, we selected first 2 compounds: wenyujinoside and 28-deglucosylchikusetsusaponin IV as candidates for further investigation (Figure 1). Docking poses of wenyujinoside, 28-deglucosylchikusetsusaponin IV, and the control (GLP-1) with GLP-1 receptor were illustrated in Figure 2. Wenyujinoside interacted with Gln97, His99, Tyr101, and Glu125 of GLP-1 receptor (Figure 2(a)). 28-Deglucosylchikusetsusaponin IV interacted with Glu97, Tyr101, and Asp122 of GLP-1 receptor (Figure 2(b)). GLP-1 interacted with Tyr101 of GLP-1 receptor (Figure 2(c)). Both the 2 candidates and the control interacted with Tyr101 of GLP-1 receptor. Thus Tyr101 was the key residue for all the 3 compounds docked with GLP-1 receptor. We investigated what kind of interaction was formed by the ligand and protein by LIGPLOT program. Wenyujinoside formed H-bond with Gln97, His99, Tyr101, and Glu125 of GLP-1 receptor. It also formed hydrophobic contact with Phe80, Asn82, Trp120, and Asp122 of GLP-1 receptor (Figure 3(a)). 28-Deglucosylchikusetsusaponin IV formed H-bond with Asp122 of GLP-1 receptor. It also formed hydrophobic contact with Phe80, Asn82, Gln97, His99, Tyr101, and Trp120 of GLP-1 receptor (Figure 3(b)). GLP-1 formed H-bond with His99 and Tyr101 of GLP-1 receptor. It also formed hydrophobic contact with Phe80, Asn82, Gln97, Asp122, Ser124, and Glu125 (Figure 3(c)). Besides Tyr101, the key residues also included Phe80, Asn82, Gln97, His99, and Asp122 for all the 3 compounds docked with GLP-1 receptor.

### 3.2. Disorder Prediction

**Figure 4:**
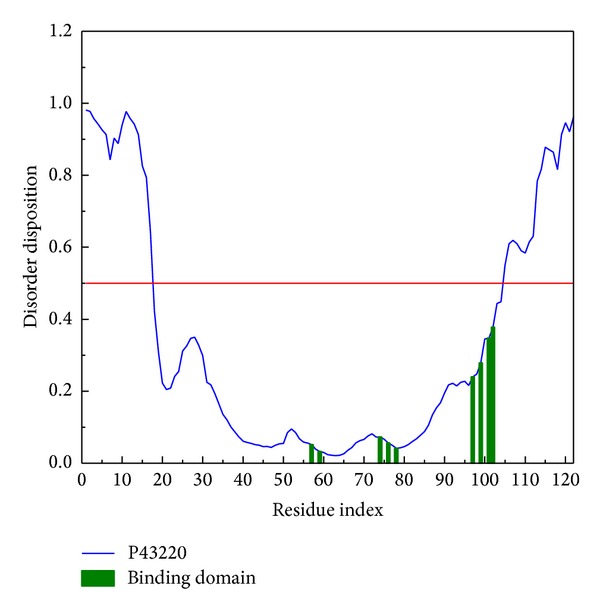
Disorder disposition of GLP1 receptor structure. The most common key residues for all the 3 compounds are in the nondisordered region (below the red line).

Besides Asp122, the other commonkey residues (Phe80, Asn82, Gln97, His99, and Tyr101) of GLP-1 receptor 3D structure for the 2 candidates and the control did not locate at the disordered region, so we could say that there was no significant influence on the shape of the main binding sites (Figure 4).

### 3.3. Molecular Dynamics (MD) Simulation

**Figure 5:**
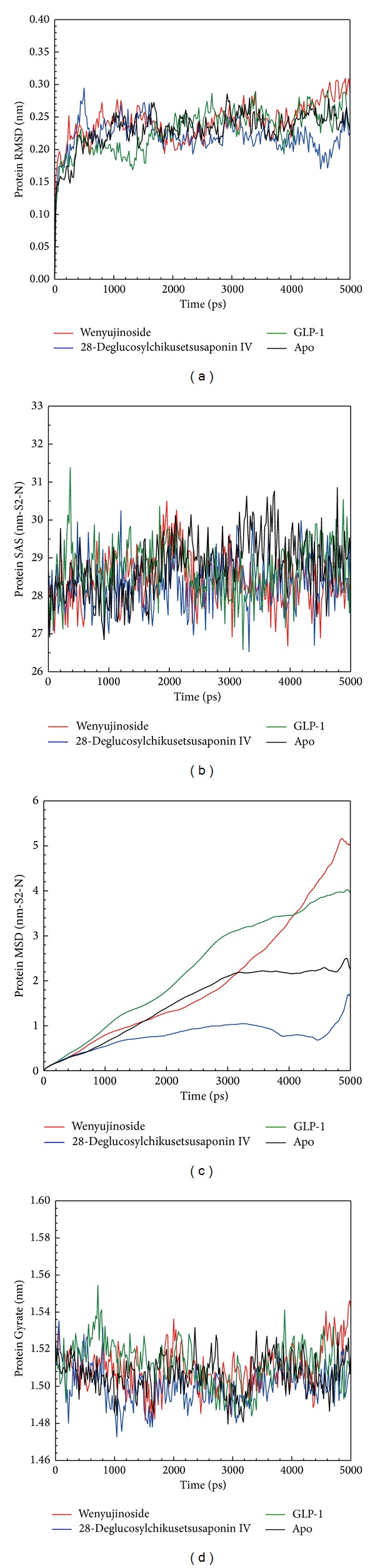
(a) RMSD, (b) SAS, (c) MSD, and (d) Gyrate for wenyujinoside, 28-deglucosylchikusetsusaponin IV, GLP1 corresponding protein, and GLP1 receptor protein alone (apo).

We drew the trajectory of RMSD to discuss the deviation of each ligand induced protein change and GLP-1 receptor protein alone during the period of MD. There was not any line graph of ligand corresponding protein RMSD that was the same as GLP-1 receptor protein alone (apo). It was evident that wenyujinoside, 28-deglucosylchikusetsusaponin IV, or the control (GLP-1) could induce conformational change of GLP-1 receptor differently (Figure 5(a)). SAS was drawn to calculate the surface area of water contact for each protein. There was not any line graph of ligand corresponding protein SAS that was the same as GLP-1 receptor protein alone (apo). It was evident that wenyujinoside, 28-deglucosylchikusetsusaponin IV, or the control could lead to surface change of GLP-1 receptor differently (Figure 5(b)). We drew the trajectory of MSD to calculate the deviation of atoms from the beginning to the end of MD. Wenyujinoside had steep rise after 3000 ps during MD. 28-Deglucosylchikusetsusaponin IV had the lowest average MSD value. We speculated that the 2 candidates could bind with GLP-1 receptor successfully despite their different patterns of MSD (Figure 5(c)). Gyrate was drawn to calculate the average distance of atoms to the center of each protein. It showed the compact degree of each protein. There was not any line graph of ligand corresponding protein Gyrate that was the same as GLP-1 receptor protein alone (apo). It was evident that wenyujinoside, 28-deglucosylchikusetsusaponin IV, or the control could induce compact change of GLP-1 receptor differently (Figure 5(d)).

**Figure 6:**
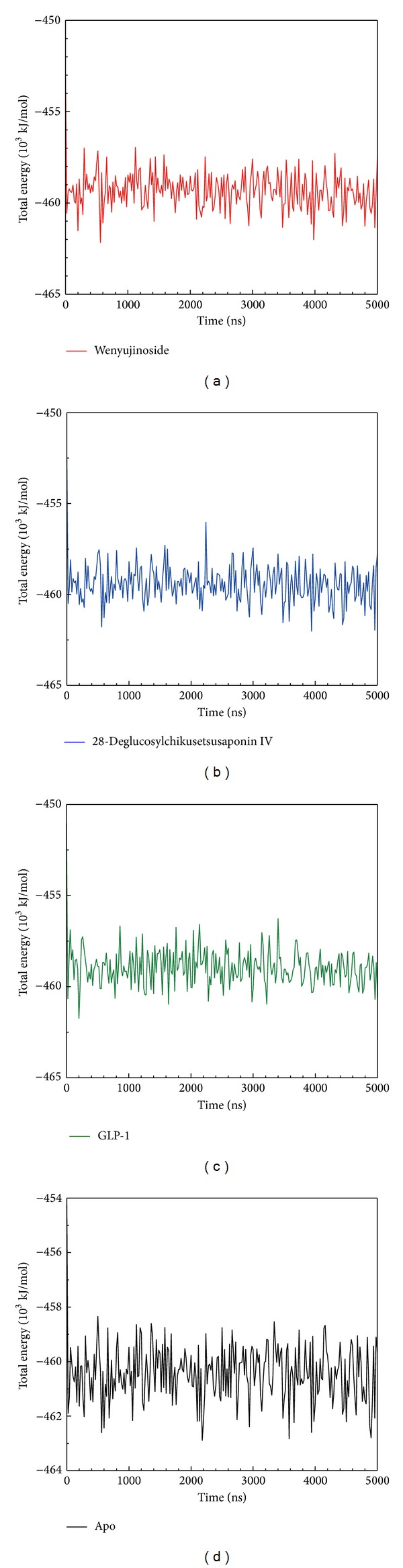
Total energy for (a) wenyujinoside, (b) 28-deglucosylchikusetsusaponin IV, (c) GLP1 corresponding protein, and (d) GLP1 receptor protein alone (apo).

The average total energy of GLP-1 corresponding protein or GLP-1 receptor alone (apo) (−461000 kJ/mol) was lower than that of wenyujinoside or 28-deglucosylchikusetsusaponin IV corresponding protein (−459000 kJ/mol) (Figure 6).

**Figure 7:**
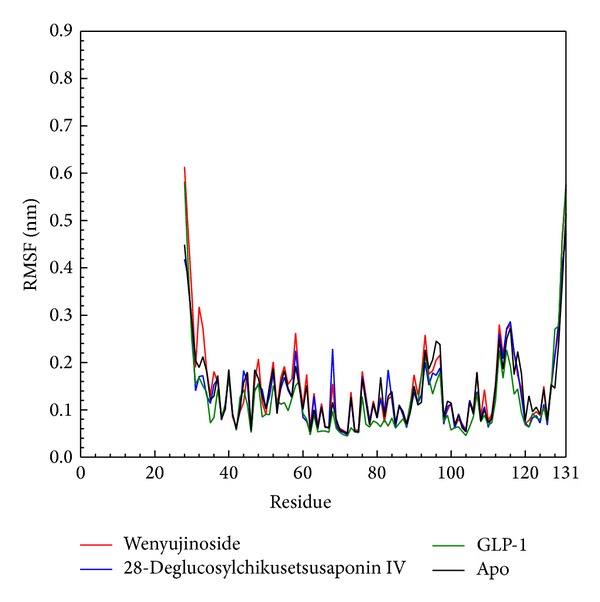
Root mean square fluctuation (RMSF) for wenyujinoside, 28-deglucosylchikusetsusaponin IV, GLP1 corresponding protein, and GLP1 receptor protein alone (apo).

**Figure 8:**
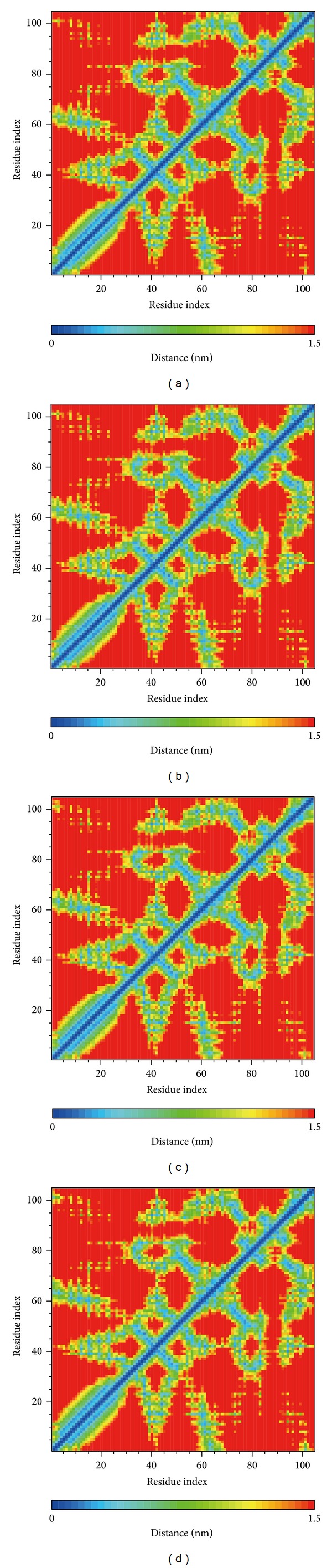
Matrices of smallest distance of residues for (a) wenyujinoside, (b) 28-deglucosylchikusetsusaponin IV, (c) GLP1 corresponding protein, and (d) GLP1 receptor protein alone.

We drew RMSF to calculate the fluctuation of every residue of the protein during MD. Wenyujinoside, 28-deglucosylchikusetsusaponin IV, GLP-1 corresponding protein, or GLP-1 receptor alone (apo) had similar line graph pattern. We speculated that when the 2 candidates and the control bound with GLP-1 receptor, every residue of their corresponding protein was under similar fluctuation (Figure 7). This finding was consistent with the figure of matrices of smallest distance of residues which was drawn to find any variation of residues distance when the ligand bound with GLP-1 receptor. There was not any apparent difference between the candidates, the control corresponding protein, and GLP-1 receptor alone (Figure 8).

**Figure 9:**
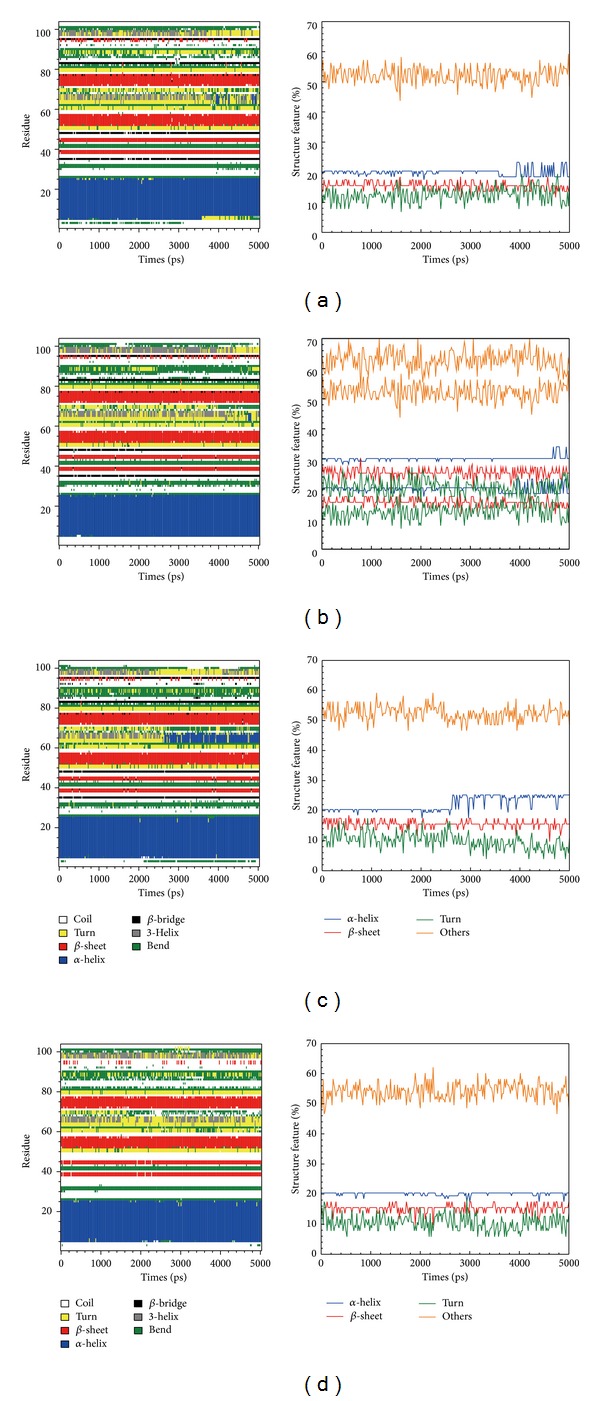
Database of secondary structure assignment (DSSP) and secondary structural feature ratio variations for (a) wenyujinoside, (b) 28-deglucosylchikusetsusaponin IV, (c) GLP1 corresponding protein, and (d) GLP1 receptor protein alone.

The figures of DSSP and secondary structural feature ratio variations were drawn to discuss the structural component change when the protein bound with the ligand. In contrast with GLP-1 receptor alone, the corresponding protein of both candidates and the control had similar finding. The ratio of *α*-helix was smooth originally, but the ratio became larger fluctuation during the late stage of MD. We speculated that activation of GLP-1 receptor followed the structural component change when it bound with the correct ligand (Figure 9).

**Figure 10:**
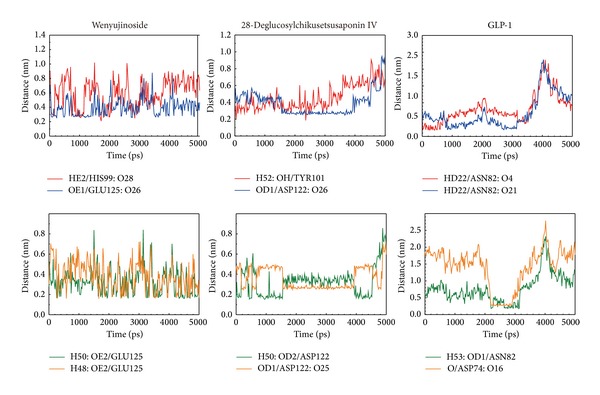
Distance of hydrogen bonds between wenyujinoside, 28-deglucosylchikusetsusaponin IV, GLP1, and essential amino acids of GLP1 receptor.

To observe the binding force of the ligand and protein, we utilized distance of H-bond between the ligand and essential amino acids. The O28 of wenyujinoside formed H-bond with His99 at early and middle of MD. The O26 of wenyujinoside formed H-bond with Glu125 at most stage of MD. The H48 and H50 of wenyujinoside also formed H-bonds with Glu125 at most stage of MD. The H52 of 28-deglucosylchikusetsusaponin IV formed H-bond with Tyr101 at early and middle stages of MD. The O26 of 28-deglucosylchikusetsusaponin IV formed H-bond with Asp122 at middle stage of MD. The H50 and O25 of 28-deglucosylchikusetsusaponin IV formed H-bonds with Asp122 at middle stage of MD too. The O4 and O21 of GLP-1 formed H-bonds with Asn82 at early and middle stages of MD, respectively. The H53 and O16 formed H-bonds with Asn82 and Asp74 at middle stage of MD, respectively (Figure 10).

**Figure 11:**
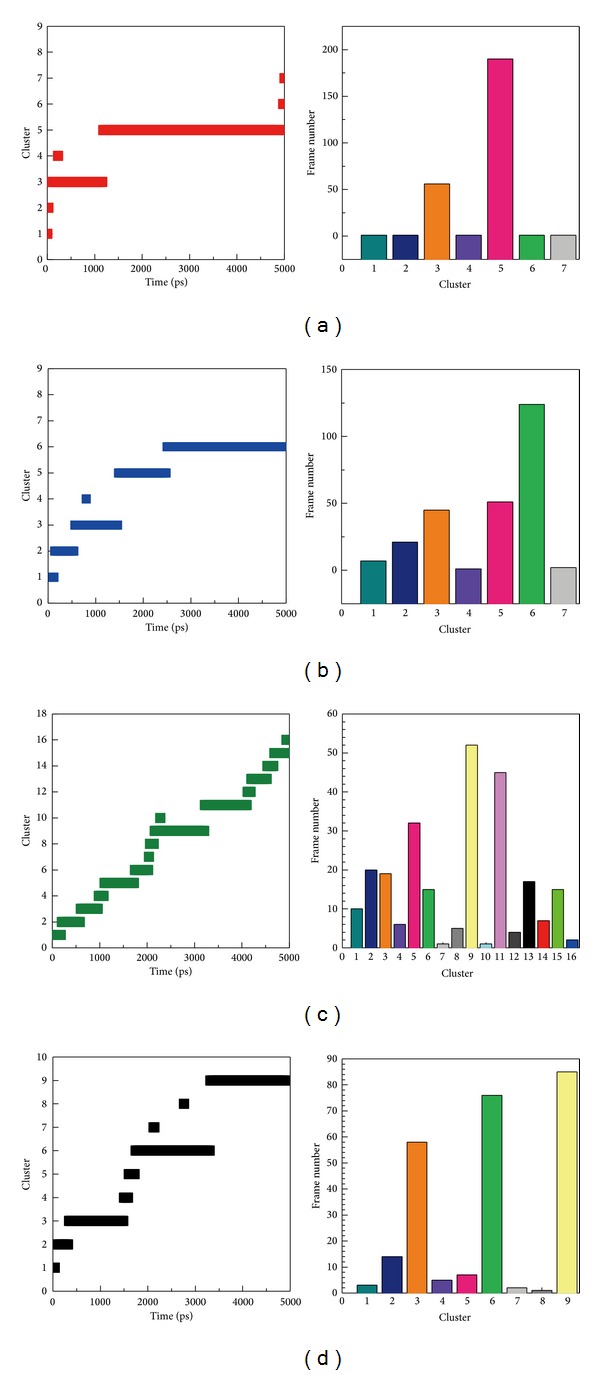
Cluster analysis for (a) wenyujinoside, (b) 28-deglucosylchikusetsusaponin IV, (c) GLP1 corresponding protein, and (d) GLP1 receptor protein alone.

We illustrated cluster analysis to point out the representative structure of protein during MD. The representative structure of wenyujinoside corresponding protein was cluster 5 from 1100 to 4940 ps. The frame number was the most of all the 7 clusters. The representative structure of 28-deglucosylchikusetsusaponin IV corresponding protein was cluster 6 from 2500 to 4920 ps. The representative structure of GLP-1 corresponding protein was cluster 9 and 11 from 2000 to 4200 ps. The representative structure of GLP-1 receptor alone was 6 and 9 from 1600 to 5000 ps (Figure 11).

**Figure 12:**
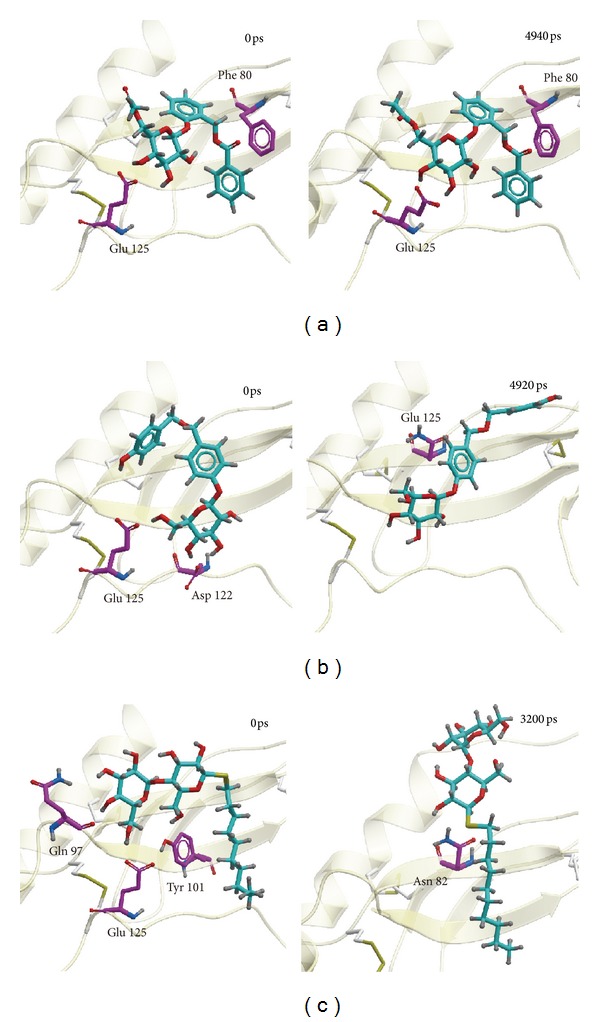
Docking poses of MD. (a) Wenyujinoside, (b) 28-deglucosylchikusetsusaponin IV, and the control: (c) GLP1.

Docking poses of MD were drawn according to integrating the figure of RMSD and the representative cluster of cluster analysis. The first picture was intercepted at 0 ps of MD for all the 3 compounds. The second picture was intercepted at 4940, 4920, and 3200 ps for wenyujinoside, 28-deglucosylchikusetsusaponin IV, and GLP-1, respectively (Figure 12). For wenyujinoside, it formed connection with Phe80 and Glu125 at 0 ps. It also formed connection with the same residues of GLP-1 receptor at 4940 ps (Figure 12(a)). For 28-deglucosylchikusetsusaponin IV, it formed connection with Asp122 and Glu125 at 0 ps. However, it only formed connection with Glu125 of GLP-1 receptor at 4920 ps (Figure 12(b)). For GLP-1, it formed connection with Gln97, Tyr101, and Glu125 at 0 ps. However, it formed connection with Asn82 instead (Figure 12(b)).

### 3.4. Ligand Pathway

**Figure 13:**
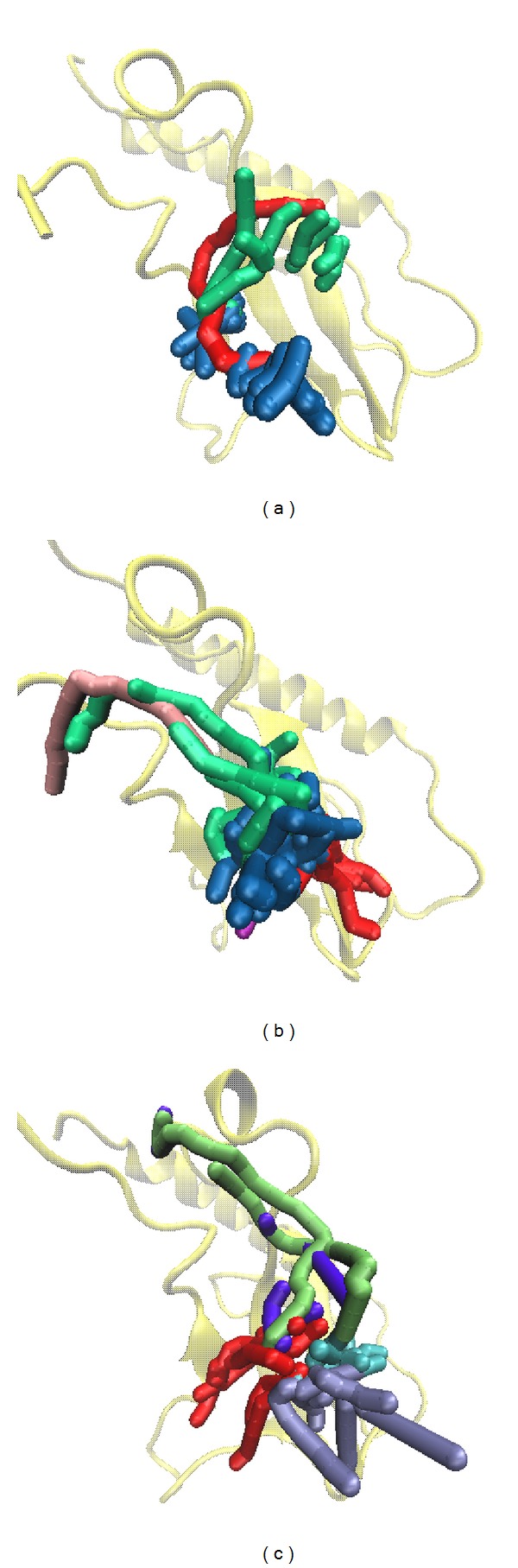
3D simulation of ligand pathway for (a) wenyujinoside, (b) 28-deglucosylchikusetsusaponin IV, and (c) GLP1 bound with GLP1 receptor protein.

3D simulation of ligand pathway was drawn to analyze all possible pathways when the ligand bound with GLP-1 receptor. All the 3 compounds had different pathways. Wenyujinoside, 28-deglucosylchikusetsusaponin IV, and GLP-1 had 3, 4, and 3 possible pathways, respectively. Thus, we could conclude that all the 3 compounds had common binding sites, but they had different pathways when they bound with GLP-1 receptor (Figure 13).

## 4. Conclusion

Diabetes mellitus (DM) and obesity are inseparable modern diseases. Excessive body mass index (BMI) increases the risk of DM. DM can cause many acute and chronic complications. Early diagnosis and adequate treatment are very important. Incretin can decrease blood glucose level. Glucagon-like peptide 1 (GLP-1) is a new, advancing agent for management of DM. However, GLP-1 will be hydrolyzed soon by the enzyme dipeptidyl peptidase-4 (DPP-4). In this study, we tried to design suitable small molecular drugs for GLP-1 receptor agonist from the world's largest TCM Database@Taiwan. Based on docking results of virtual screening, we selected 2 TCM compounds, wenyujinoside and 28-deglucosylchikusetsusaponin IV, for further investigation. Wenyujinoside was mainly extracted from* Curcuma wenyujin*. 28-Deglucosylchikusetsusaponin IV was mainly extracted from* Codonopsis convolvulacea* var. forrestii. GLP-1 was assigned as the control compound. Phe80, Asn82, Gln97, His99, Tyr101, and Asp122 were the common key residues for all the 3 compounds docked with GLP-1 receptor. Based on the figures of RMSD, SAS, MSD, and Gyrate, we could conclude that all the 3 compounds induced different conformational change of GLP-1 receptor. Interestingly, from the view of individual residues, there was not any apparent difference between the 3 compounds in the figures of RMSF and matrices of smallest distance of residues. In the figure of DSSP and secondary structural feature ratio variations, we concluded that activation of GLP-1 receptor followed the structural component change when it bound with the correct ligand. We could say that MD simulation was dynamic condition according to the figures of distance of H-bond, cluster analysis, and docking poses of MD. Finally, we concluded that all the 3 compounds could bind and activate GLP-1 receptor. Wenyujinoside and 28-deglucosylchikusetsusaponin IV were the TCM compounds that could be GLP-1 receptor agonists.

## Figures and Tables

**Table 1:** Top 10 candidates of scoring function based on TCM Database@Taiwan screening.

Name	-PLP2	-PLP1	-PMF
Wenyujinoside	81.72	79.95	165.82
28-Deglucosylchikusetsusaponin IV	70.99	66.94	149.93
(6aR_11aR)-9_10-Dimethoxypterocarpan-3-O-beta-D-glucoside	70.59	71.5	165.58
Formononetin-7-O-beta-D-glucoside	70.39	71.07	156.71
(3R_5S)-3-Acetoxy-5-hydroxy-1_7-bis(4-hydroxy-3-methoxyphenyl)heptane	70.09	68.57	158.18
Alpha-caryophyllene	70.05	73.91	162.77
Ononin	70.03	73.04	157.81
(5S)-5-Acetoxy-1_7-bis(4-hydroxy-3-methoxyphenyl)heptan-3-one	68.63	72.86	152.6
(5R)-5-Hydroxy-1-(4-hydroxy-3-methoxyphenyl)7-(4_5-dihydroxy-3-methoxyphenyl)-3-	67.31	61.05	152.3
3-O-(2_E_4_Z)-decadienoylingenol	66.07	65.93	173.24

GLP1	64.52	56.19	152.39

PLP: piecewise linear potentials. PMF: potential of mean force.
